# CT-based radiomics for predicting breast cancer radiotherapy side effects

**DOI:** 10.1038/s41598-024-70723-w

**Published:** 2024-08-29

**Authors:** Óscar Llorián-Salvador, Nora Windeler, Nicole Martin, Lucas Etzel, Miguel A. Andrade-Navarro, Denise Bernhardt, Burkhard Rost, Kai J. Borm, Stephanie E. Combs, Marciana N. Duma, Jan C. Peeken

**Affiliations:** 1 Department of Radiation Oncology, Klinikum Rechts der Isar, Technische Universität München, Ismaninger Straße 22, 81675 Munich, Germany; 2 Department of Informatics, Bioinformatics and Computational Biology—i12, Technische Universität München, Boltzmannstr. 3, 85748 Munich, Germany; 3 Institute of Organismic and Molecular Evolution, Johannes Gutenberg University Mainz, Hanns-Dieter-Hüsch- Weg 15, 55128 Mainz, Germany; 4 Deutsches Konsortium für Translationale Krebsforschung (DKTK), Partner Site Munich, 69120 Heidelberg, Germany; 5 Department of Radiation Sciences (DRS), Institute of Radiation Medicine (IRM), Helmholtz Zentrum, 85764 München, Germany; 6 Department of Radiation Oncology, Helios Clinics of Schwerin - University Campus of MSH Medical School Hamburg, Schwerin, Germany; 7 Department for Human Medicine, MSH Medical School Hamburg, Hamburg, Germany

**Keywords:** Radiomics, Machine learning, Breast cancer, Computed tomography, Radiotherapy, Side effects, Skin inflammation, Moist cells epitheliolysis, Edema, Breast cancer, Machine learning, Breast cancer, Oedema, Skin manifestations, Breast cancer

## Abstract

Skin inflammation with the potential sequel of moist epitheliolysis and edema constitute the most frequent breast radiotherapy (RT) acute side effects. The aim of this study was to compare the predictive value of tissue-derived radiomics features to the total breast volume (TBV) for the moist cells epitheliolysis as a surrogate for skin inflammation, and edema. Radiomics features were extracted from computed tomography (CT) scans of 252 breast cancer patients from two volumes of interest: TBV and glandular tissue (GT). Machine learning classifiers were trained on radiomics and clinical features, which were evaluated for both side effects. The best radiomics model was a least absolute shrinkage and selection operator (LASSO) classifier, using TBV features, predicting moist cells epitheliolysis, achieving an area under the receiver operating characteristic (AUROC) of 0.74. This was comparable to TBV breast volume (AUROC of 0.75). Combined models of radiomics and clinical features did not improve performance. Exclusion of volume-correlated features slightly reduced the predictive performance (AUROC 0.71). We could demonstrate the general propensity of planning CT-based radiomics models to predict breast RT-dependent side effects. Mammary tissue was more predictive than glandular tissue. The radiomics features performance was influenced by their high correlation to TBV volume.

## Introduction

Breast cancer is the leading form of invasive cancer in women, accounting for the most significant proportion of cancer cases worldwide^1,2^. Approximately 14% of women are affected by breast cancer, making it a prevalent health concern^3^. Radiation therapy (RT) constitutes the standard of care after breast-conserving surgeries for most patients^4^.

Radiomics, a field dedicated to extracting quantitative features from medical imaging such as computer tomography (CT) paired with machine learning (ML), shows great potential in cancer research^5^. Radiomics provides a powerful foundation for the integration of ML techniques in cancer research. By extracting quantitative features from medical images, radiomics enables the generation of high-dimensional data, which can then be utilized by computational models to create predictive models for clinical or biological endpoints^6–8^.

In the context of mammary carcinoma, radiomics has been widely applied to predict survival, disease progression, treatment response, molecular aberrations, and the detection of metastases or areas of infiltrative tumor^9–16^. Nevertheless, the application of radiomics analysis for accurately predicting non-tumor response to RT remains limited. Earlier research has explored the possibility of predicting RT-related side effects, including xerostomia and pneumonitis, or pain response to palliative RT^17–19^.

Several similar studies have investigated the use of ML and various types of imaging data to predict RT side effects in breast cancer patients. Research utilizing dosiomics features extracted from CT images managed to accurately predict acute skin toxicity^20^. Another study using electron density and biologically effective dose radiomics effectively predicted late radiation-induced subcutaneous fibrosis^21^. Additionally, a comprehensive review of ML models analyzed RT-induced complications across multiple cancer sites, including breast cancer^22^. Collectively, these studies emphasize the growing interest in using ML and imaging data to mitigate RT side effects.

The objective of this study was to develop a statistically reliable assessment of the predictive capability of radiomics features to predict the most prevalent RT side effects of moist epitheliolysis as a surrogate for skin inflammation and edema based on the total breast volume (TBV) and glandular tissue (GT).

## Materials and methods

### Clinical data collection and curation

The dataset consisted of 252 breast cancer patients who underwent radiotherapy between 2012 and 2016 in the Rechts der Isar university hospital of the technical university of Munich (TUM). For the patient data acquired at TUM, retrospective analysis of patient records and data is generally allowed following Article 27 of the Bavarian Hospital act (Bayerisches Krankenhausgesetz) from the Landeskrankenhausgesetz des Freistaates Bayern. Informed consent for treatment was obtained from every patient. Institutional Review Board (IRB) was acquired from the review board of TUM (reference number 466/16 s. Clinical variables were defined based on a literature review on known clinical predictors from previous publications. Moreover, variables were selected based on broad availability of data that hindered the assessment of other predictive factors^23,24^: smoker status, chemotherapy received, radiotherapy boost, the maximum prescribed radiation dose in equivalent dose at 2 Gy (EQD2, $\:\propto\:/\beta\:$ = 3), TBV, and the two targets of prediction: (i) moist cell epitheliolysis as surrogate for common terminology criteria for adverse effects (CTCAE) grade 2 skin inflammation^25^ (33 positive cases; referred henceforth simply as moist epitheliolysis); and (ii) presence of any edema (26 positive cases).

### Radiomics data collection and curation

Prior to RT treatment, planning CT images of the breast were conducted. Figure S1 shows que acquisition parameters for these CT images. Exclusion criteria encompassed breast implant and mastectomy cases. Two separate volume of interest (VOI) definitions were segmented, creating two radiomics cohorts: TBV, containing radiomics information from the whole breast tissue; and glandular tissue (GT), which contained radiomics information only from this tissue. Patient outcome assessment was performed retrospectively by a medical student after thorough teaching by a radiation oncologist (JCP). All methodology has been conducted in accordance to the relevant guidelines and regulations.

Segmentation of the volumes of interest was manually performed by NW, using 3D Slicer^26^. GT was defined using the fast growcut function. BSpline interpolation was used to perform isotropic resampling to obtain a voxel size of 1 × 1 × 1 mm. Image discretization was carried out with a fixed bin width of 10. Laplacian of Gaussian filtering was used for image reconstruction (Sigma values of 1.0, 2.0, 3.0, 4.0 and 5.0).

**Fig. 1:**
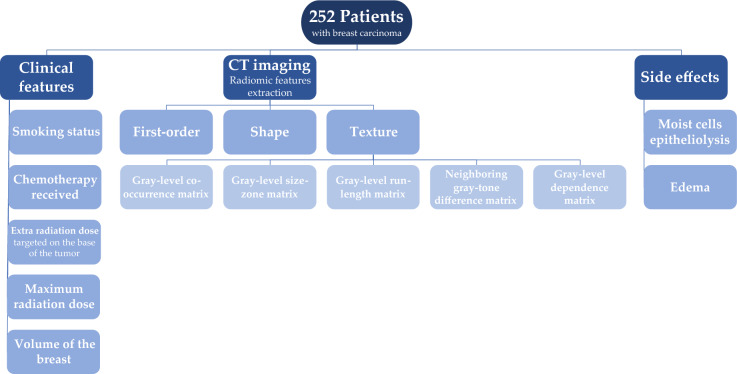
Patient and data flowchart. In the left and central branches, the clinical and radiomics features can be found, respectively. The right branch shows the three RT side effects used as prediction targets.

Radiomics features were extracted and filtered from the CT images and both segmentations using the Python library PyRadiomics^27^ (version 3.0.1; Python version 3.8.10). A total of 104 features were obtained for each of the radiomics cohorts, which included first-order, shape, and texture features (the latter is composed of “gray-level co-occurrence matrix”, “gray-level size-zone matrix”, “gray-level run-length matrix”, “neighboring gray-tone difference matrix”, and “gray-level dependence matrix” features). Figure 1 shows a diagram of the clinical and radiomics features and side effects collection process from the patients. Further, Fig. S2 shows the distribution of patients across all clinical features and side effects measured.

### Feature pre-processing and hyperparameter optimization

Repeated nested cross-validation was employed to train and validate the models. Normalization of the radiomics features was performed using min-max normalization, in order to conserve the original distribution in the [0, 1] range.

For each cohort, the most interesting features were selected and evaluated in two different ways: the first one, with a double Spearman rank correlation test, first within each dataset with a cut-off value of 0.9 to remove redundant features; and then towards each side effect prediction target, in order to keep the most relevant features. The second option was selecting features using minimum redundancy-maximum relevance (MRMR; version 1.0.2), which incorporates both tests in a single step^28^. In both cases, an estimation of the information density and, therefore, of the number of features to select, was made using Principal Component Analysis (PCA). For the TBV radiomics feature set, an average of 23 and 39 features were selected when using MRMR and a double Spearman rank correlation test, respectively. For the GT radiomics feature set, on the other hand, an average of 26 and 44 features were selected when using each of the feature selection techniques, respectively.

Before finding the optimal hyperparameter values, the class imbalance of the different side effect prediction targets was corrected depending on the level of disproportion. Moist epitheliolysis and edema had a ratio of 6.64:1 and 8.69:1 of negative to positive class sizes, respectively, and were therefore corrected using a combination of synthetic minority over-sampling technique (SMOTE; *imbalance-learn* library version 0.11.0)^29^ to a ratio of 2:1, and random under-sampling of the majority class to a ratio of 1.25:1. The choice of ratios for each step was made to find a balance between avoiding excessive oversampling and losing too many samples while undersampling. Balanced accuracy (BA) was the metric used as optimization criteria for the values of the hyperparameters, capable of handling the small remainder of class imbalances. Hyperparameter optimization was conducted using an exhaustive grid search, where all combinations of hyperparameter values are tested in the validation set of the innermost fold until the optimal values are found.

### Machine learning modeling

Four ML algorithms were implemented and evaluated: logistic regression (LR), used for its simplicity and efficiency in binary classification tasks with a low feature set dimensionality^30,31^; least absolute shrinkage and selection operator (LASSO), a variant with an optimizable regularization term that can potentially better handle imbalanced datasets^32^; support vector machine (SVM), a high flexibility algorithm thanks to the implementation of multiple kernels and explore non-linear relationships in the data^33^; and random forest classifier (RF), an ensemble learning, decision tree-based method that is more robust to overfitting effects^34^. All models were imported from the python library scikit-learn (version 1.0.2)^35^. These models were contrasted against clinical model baselines.

**Fig. 2:**
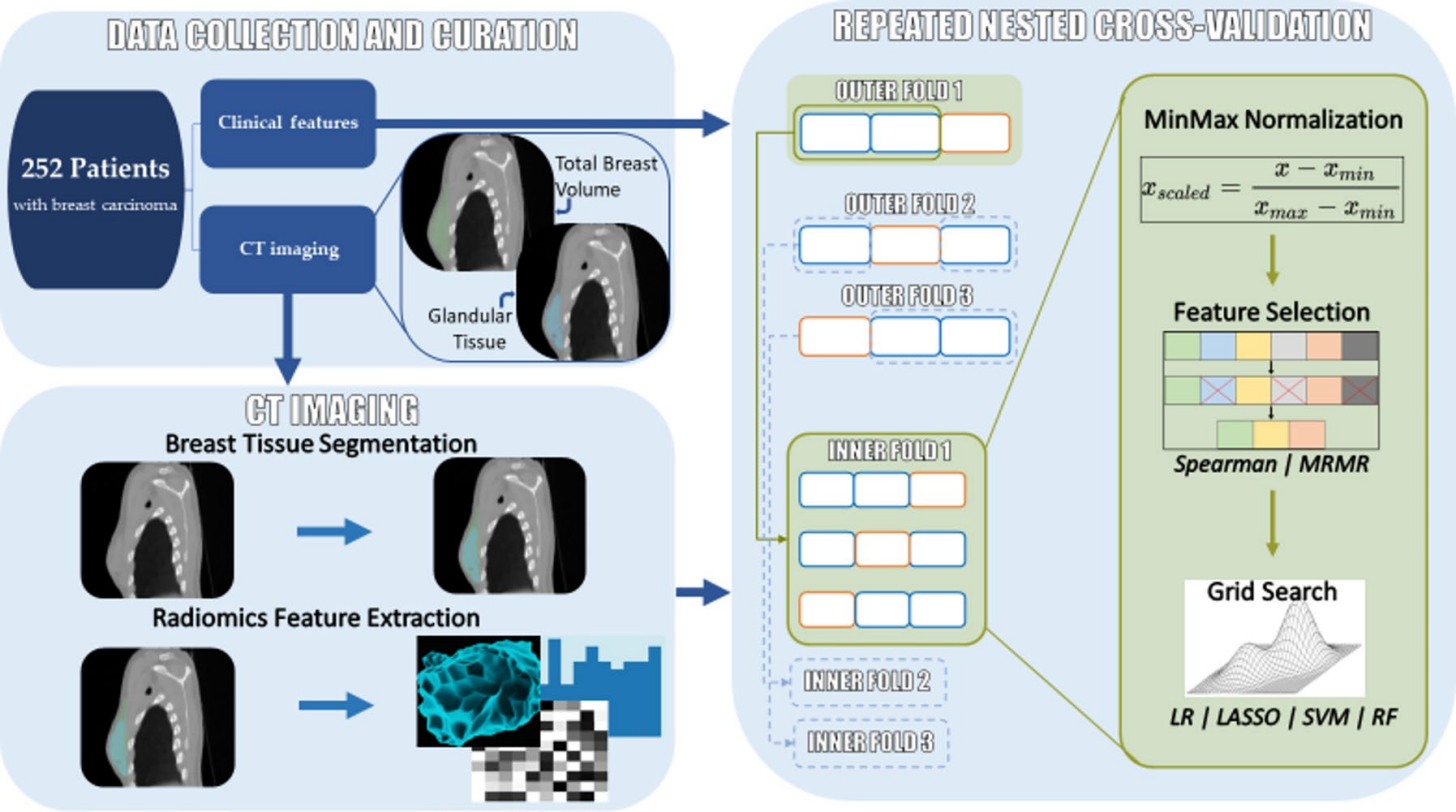
Workflow of the pipeline used in the study to analyze both clinical and radiomics data. On the left half of the workflow: clinical features were obtained from all patients with CT imaging available, the respective VOIs (TBV and GT) were segmented, and the subsequent radiomics features extracted. On the right half of the workflow: for each evaluated dataset, a 50-repeat nested cross-validation was performed. Within the inner fold normalization, feature selection and an exhaustive grid search for optimal hyperparameters was performed.

After comparing the four model types for each of the radiomics cohorts and feature selection types, the best models were retrained and optimized adding clinical data in order to assess whether a combined model yields a better performance in predicting the presence of any side effect. The workflow followed by the ML pipeline is shown in Fig. 2. In addition, larger reference images of the respective VOIs can be seen in Fig. S1.

Feature selection has been analyzed for all relevant models, estimating a score based on the feature importance assigned by the models and how often each feature was selected. The resulting score is calculated as $\:Score=\:Feature\:Importance/[\left(n+1\right)-m]$, where *n* is the number of models, and *m* is the number of times the feature has been chosen.

Finally, the correlation between the breast volume and the prediction probability of the best model has been analyzed to study the overall impact of the breast volume in the predictive value of radiomics features. An additional model was evaluated where radiomics features that highly correlated to the breast volume were excluded (Spearman correlation higher than 0.8), using the best performing configuration. The objective was to assess the impact of volume-correlated features on the performance of radiomics models.

### Statistical analysis

Training and validation of the different models were performed using 50 repetitions of nested cross-validation (5 outer folds, 4 inner folds). This resampling technique provides additional statistical robustness, resulting in 250 final models that were aggregated to the final test results.

In order to gather more information from the radiomics features, PCA was employed as an estimation of the information density within this dataset. The variance retention by the components of PCA was used to understand the intrinsic dimensionality of our dataset. However, since the components generated by PCA are a different combination from the original features and, generally, more packed, these components should not be used as a feature selection replacement, but as an estimation. The reason behind it is the inherent added difficulty of tracing the feature importance back to the original features.

In the inner fold of the nested cross-validation normalization, feature selection and class imbalance correction were applied, in order to avoid data leakage from any training split to the validation (inner fold) or test splits (outer fold).

One of the two feature selection techniques mentioned in this study is the use of a double Spearman rank correlation test. This approach is intended to optimize feature selection by addressing redundancy and relevance in two distinct steps. First, redundancy is removed so that features that do not provide additional information are eliminated. Second, the Spearman rank correlation test is applied again comparing the dataset and the predictor, selecting instead the features that are most relevant to the prediction target.

The performance of the aggregated models was measured using a combination of metrics: BA, F1, precision, recall, specificity, area under the receiver-operator curve (AUROC) and Matthew’s correlation coefficient (MCC). Metrics are given with 1.96 standard errors for a confidence interval of 95%. ROC curves were also used to evaluate the trade-off between the sensitivity and specificity across different decision thresholds, and to assess the discrimination power between classes of each of the models.

## Results

We evaluated the possibility of predicting side effects of RT in breast cancer (moist cells epitheliolysis as a surrogate for skin inflammation and edema) based on the total breast volume (TBV), glandular tissue (GT) and using clinical features. Table 1 summarizes the results that are shown throughout this section. The feature importance was calculated for the best performing radiomics and clinical models (Table 2 and Table S8, respectively).

**Table 1:** Summary of the best AUROC performances for a given feature set used for training and side effect predicted.

Side effect	TBV	GT	Clinical
Moist epitheliolysis	0.74 ± 0.01	0.65 ± 0.01	0.70 ± 0.01
Edema	0.53 ± 0.02	0.55 ± 0.01	0.53 ± 0.02

### Side effect prediction

**Fig. 3:**
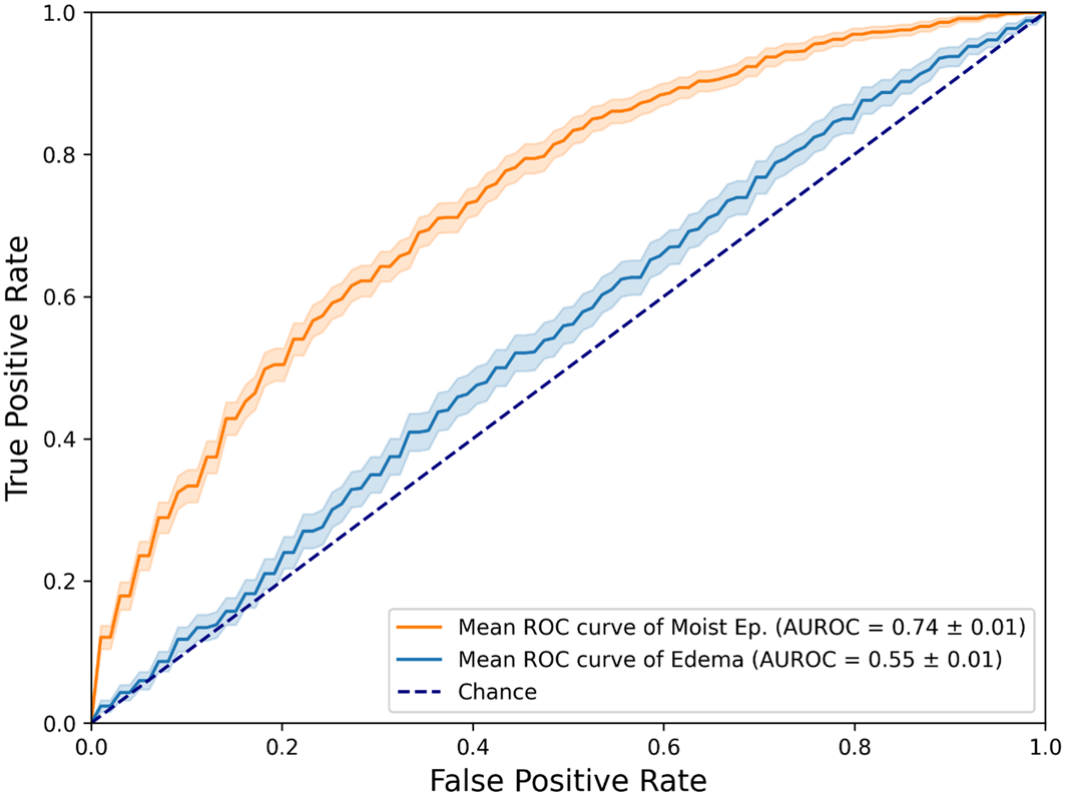
Test ROC curves of the best performing models for each of the side effects predicted. Moist cells epitheliolysis: LASSO classifier trained on TBV radiomics features, selected by MRMR. Edema: LASSO classifier trained on GT radiomics features, selected by Spearman rank correlation.

The ROC performance of the best trained models to predict both side effects can be seen in Fig. 3. More scores regarding the comparison of side effects as the prediction target can be seen in Table S2. In addition, the calibration curve of the best performing radiomics model is shown in Fig. S5.

While the edema models performed only slightly above random (best AUROC value of 0.55), both radiomics feature sets have shown a notable predictive value towards moist cells epitheliolysis using the LASSO classifier: an AUROC of 0.74 when using TBV, and an AUROC of 0.65 when training a RF on the GT radiomics feature set, whose features were selected using MRMR. Therefore, models trained to predict moist cells epitheliolysis perform better than predicting edema regardless of the feature selection technique, ML algorithm, or the training radiomics feature set used.

**Fig. 4:**
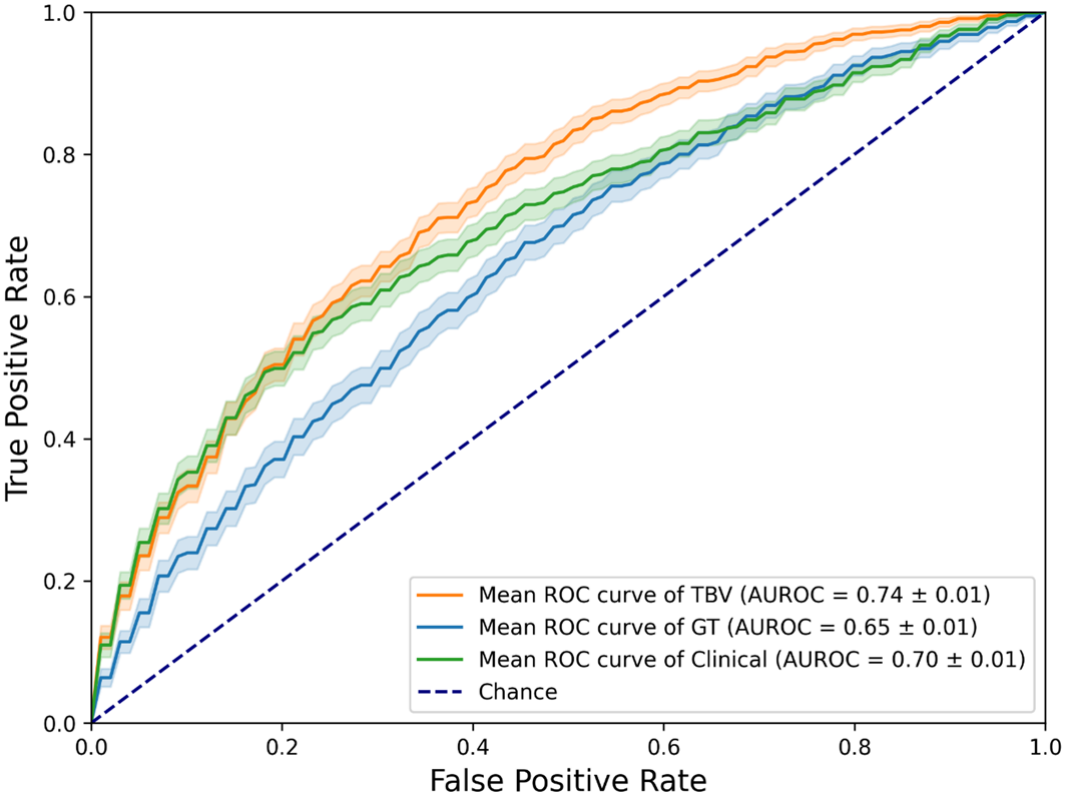
Test ROC curves of the best performing models depending on the training data used for moist epitheliolysis. TBV radiomics features: LASSO classifier, with features selected by MRMR, predicting moist cells epitheliolysis. GT radiomics features: RF classifier, with features selected by MRMR, predicting moist cells epitheliolysis. Clinical baseline: LR classifier, with features selected by Spearman rank correlation, predicting moist cells epitheliolysis.

The ROC performance of both radiomics cohorts, with the clinical features as baseline, are shown in Fig. 4. The clinical model achieved an AUROC of 0.7. More scores regarding the comparison of the predictive power of each radiomics feature set and the clinical baseline can be seen in Table S3. Only the best performing ML algorithms are being shown, according to the evaluation of the four types of models (shown in Table S4). An additional analysis of the best feature selection approach has been made (Table S5).

### Combined modelling

**Fig. 5:**
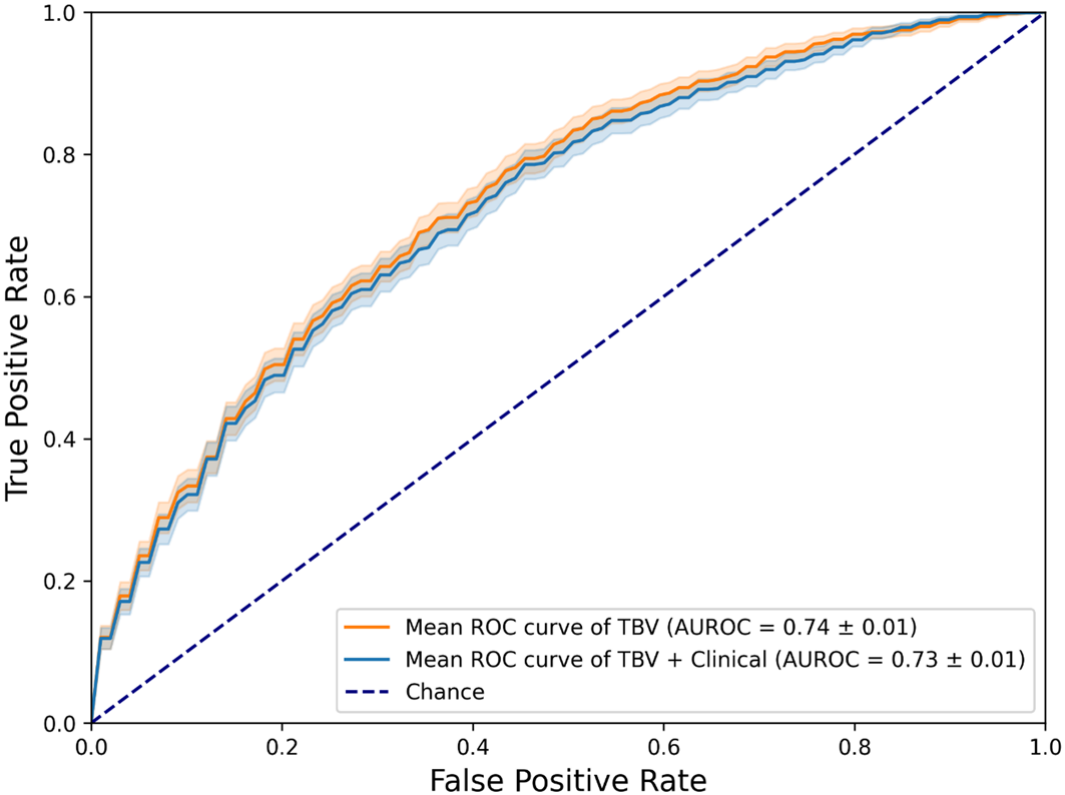
Test ROC curves of the best performing model trained on a single feature set and the best performing model trained on a combined feature set. Best single feature set model: LASSO classifier trained on TBV radiomics features, selected by MRMR, predicting moist cells epitheliolysis. Best combined feature set model: LASSO classifier trained on a combination of TBV and clinical features, selected by MRMR, predicting moist cells epitheliolysis.

Figure 5 shows the best performing models using combined datasets of either radiomics feature sets and clinical features. More scores regarding the comparison of the combinations of radiomics feature sets with clinical features, and their respective predictive performance, can be found in Table S6. When combining TBV radiomics features with clinical ones, LASSO performed best when predicting moist cells epitheliolysis (AUROC of 0.73) although without any overall improvement. RF performed best when predicting edema (AUROC of 0.53), though just above random.

### Feature importance

Table 2 shows the feature importance scores for the best models. To account for both the importance score and the frequency by which a feature was chosen, we computed a score that was the product of these values and ranked the features accordingly.

**Table 2:** Top 15 feature importance report of the best performing model: a LASSO classifier trained on TBV radiomics features, selected by MRMR, and predicting moist cells epitheliolysis as a surrogate for skin inflammation. This report shows how often a feature has been chosen out of the 250 iterations (% chosen), the feature importance value given by the model (importance; LASSO coefficients), and a score encompassing the feature importance value and how often that feature was selected (product of these two values).

Feature type - name	% chosen	Importance	Score
Shape - Maximum D Diameter Column	99.6	4.30	4.29
Shape - Least Axis Length	100	2.39	2.39
Glcm - Imc	96.4	1.59	1.53
Shape - Surface Area	95.2	1.39	1.32
Shape - Flatness	88.8	1.44	1.28
Glszm - Gray Level Non-Uniformity	98.4	1.05	1.03
Glrlm - Run Length Non-Uniformity	99.6	1.00	0.99
Shape - Maximum D Diameter	53.6	1.83	0.98
Glszm - Size Zone Non-Uniformity	66.4	1.47	0.97
Glrlm - Gray Level Non-Uniformity	93.6	1.01	0.94
Shape - Major Axis Length	77.6	1.20	0.93
Shape - Maximum D Diameter Slice	35.6	2.33	0.83
Firstorder - Energy	96.4	0.85	0.82
Gldm - Dependence Variance	84	0.96	0.81
Shape - Maximum D Diameter Row	65.2	1.23	0.80

From the list of the 15 most predictive features, more than half of them belong to the shape type, confirming that planar and volumetric information has a significant influence on the performance of oncological ML models^36–38^.

### Predictive influence of the total breast volume

The influence of the volume of the whole breast on the prediction quality of the models has been further analyzed. A logistic regression model has been trained only on TBV breast volume, with an AUROC of 0.75 ± 0.01, performing similarly to the best model trained on all TBV radiomics features.

Over all 250 runs, there was a median Spearman correlation coefficient of 0.82 between TBV breast volume and the best radiomics model. Figure S3 shows the Spearman’s correlation distribution between the TBV breast volume and the prediction probabilities of the best performing model. The distribution of the respective p-values can be seen in Fig. S4. The p-values of their correlation to the prediction probabilities of the model were significant (*p* < 0.05) in 243 runs.

The predictive influence of the breast volume has been evaluated by retraining the best performing model, but excluding all features with a Spearman correlation coefficient higher than 0.8. These results can be seen in Table S7. With an AUROC of 0.71, performance has slightly but significantly decreased (from an AUROC of 0.74), confirming an effect of the breast volume on the performance of these radiomics models.

## Discussion

In this study, we analyzed the relevance of CT-based radiomics to predict two common RT side effects: epitheliolysis of moist cells as a surrogate for skin inflammation; and edema, using a statistically robust pipeline. The best prediction model was a LASSO classifier that was trained on radiomics features from the TBV and selected using MRMR, predicting moist cells epitheliolysis. This model achieved a moderate discriminatory power with an AUROC of 0.74. Clinical features alone or in combination with radiomics did not significantly improve predictive performances.

In contrast, edema was more difficult to predict with a performance level just above random (AUROC score of 0.55 for the best model). The best radiomics model for moist cell epitheliolysis was largely correlated to the TBV volume which itself showed the same reasonable predictive performance with an AUROC of 0.74.

These results have uncovered the previously known fact that radiomics features are largely correlated with the size of the VOI^39,40^. Eliminating volume-correlated features slightly mitigated the performance of the radiomics model (AUROC of 0.71 from 0.74). As consequence, radiomic features do carry relevant information for the prediction of radiotherapy side effects. However, these features are less predictive than TBV volume.

The analysis of the importance of other features revealed several logical patterns. First, shape features appeared to be the most influential ones, indicating that geometrical features play a dominant role in predicting RT-dependent side effects. Maximum D Diameter Column being the most influential feature supports this idea, implying that larger tumors or more irregular tumors may cause more adverse effects to RT due to how the dose distribution is made, and how it affects the neighboring tissue. Further, the presence of multiple gray level types of features suggests that the heterogeneity of the tumor tissue is another significant factor, possibly due to how different types of tissues may react to RT, and the side effects that appear as a cause of this non-uniformity^41,42^.

Naturally, the given radiation dose is a decisive factor for development or RT-dependent side effects. The dose was part of the clinical prediction model achieving a decent predictive performance albeit inferior to the TBV volume. In fact, the radiation doses given were largely similar, yielding low variability and thus predictive value. Moreover, this cohort was solely treated with normofractionated RT (conventional RT dose fractionation schedule). The START B trial, however, could also demonstrate the predictive performance of breast size on physician-assessed normal tissue effects in the breast^43^.

While LASSO yielded the overall best results, all other ML algorithms have proven to be on a similar level. Only SVM has performed slightly but statistically worse, with an AUROC of 0.69 on the best configuration (compared to LASSO: AUROC of 0.74). The choice of algorithm is relevant but does not affect the performance of the model, as long as the model is optimized and properly trained. The choice of the feature selection technique had a small impact on the overall performance, managing to reduce the data dimensionality without losing much information.

This study is subject to two main limitations. The first one stems from the retrospective nature of our side effect data, deriving from past patient records, which presents a challenge to data quality. To this end, we decided to predict moist cells epitheliolysis as it constitutes a binarized endpoint describing more aggravated skin inflammation. On the other hand, the detection and extent of edema was completely dependent on the subjective physician assessment. The second limitation regards the absence of an external validation cohort for an unbiased estimation of the performance of our models. To compensate for this and have a more reliable and unswayed model performance assessment, we decided to apply a more robust resampling technique, in this case a 50-repeat nested cross-validation.

## Conclusions

To conclude, the radiomics models developed in this study have shown a reasonable prediction power towards the epitheliolysis of moist cells side effect, while clinical features yielded intermediate albeit competitive results. Adding information from the whole breast tissue, instead of just glandular tissue, achieved better results overall. The radiomics prediction probabilities were largely correlated to breast volume which remained the most predictive feature, though this correlation only affected to a small extent the prediction power of radiomics features in general. These findings, however, should be further validated on larger, more diverse and multi-centered datasets. Future studies should investigate the potential variations in RT side effects prediction using radiomics information depending on the subtype and stage of breast cancer.

### Supplementary Information

Supplementary Information.

## Data Availability

All data and code used in this research is available upon contact of the correspondence author (Óscar Llorián-Salvador, oscar.llorian-salvador@tum.de) and in concordance to the ethics committee.

## References

[1] Bray, F. *et al.* Global Cancer Statistics 2018: GLOBOCAN estimates of incidence and mortality worldwide for 36 cancers in 185 countries. *CA Cancer J. Clin.* **68**, 394–424. 10.3322/caac.21492 (2018). 30207593 10.3322/caac.21492

[2] Siegel, R. L., MIller, K. D., Wagle, N. S. & Jemal, A. Cancer statistics. *CA Cancer J. Clin.* **73**, 17–48. 10.3322/caac.21763 (2023). 36633525 10.3322/caac.21763

[3] Lin, L. *et al.* Regional, and national cancer incidence and death for 29 cancer groups in 2019 and trends analysis of the global cancer burden, 1990–2019. *J. Hematol. Oncol.* **14**, 197. 10.1186/s13045-021-01213-z (2021). 34809683 10.1186/s13045-021-01213-z PMC8607714

[4] Shah, C., Al-Hilli, Z. & Vicini, F. Advances in breast cancer radiotherapy: implications for current and future practice. *JCO Oncol. Pract.* **17**, 697–706. 10.1200/OP.21.00635 (2021). 34652952 10.1200/OP.21.00635

[5] Peeken, J. C., Wiestler, B., Combs, S. E., Image-Guided, & Radiooncology,. The potential of radiomics in clinical application. *Recent. Results Cancer Res.* **216**, 773–794. 10.1007/978-3-030-42618-7_24 (2020). 32594406 10.1007/978-3-030-42618-7_24

[6] Kumar, V. *et al.* Radiomics: The process and the challenges. *Magn. Reson. Imaging* **30**, 1234–1248. 10.1016/j.mri.2012.06.010 (2012). 22898692 10.1016/j.mri.2012.06.010 PMC3563280

[7] Desideri, I. *et al.* Application of radiomics for the prediction of radiation-induced toxicity in the IMRT era: Current state-of-the-art. *Front. Oncol.* **10**, 1708 (2020). 33117669 10.3389/fonc.2020.01708 PMC7574641

[8] Peeken, J. C. *et al.* Prognostic assessment in high-grade soft-tissue sarcoma patients: A comparison of semantic image analysis and radiomics. *Cancers* **13**, 1929. 10.3390/cancers13081929 (2021). 33923697 10.3390/cancers13081929 PMC8073388

[9] Bi, W. L. *et al.* Artificial intelligence in cancer imaging: Clinical challenges and applications. *Cancer J. Clin.* **69**, 127–157. 10.3322/caac.21552 (2019). 10.3322/caac.21552 PMC6403009 30720861

[10] Bera, K., Braman, N., Gupta, A., Velcheti, V. & Madabhushi, A. Predicting cancer outcomes with radiomics and artificial intelligence in radiology. *Nat. Rev. Clin. Oncol.* **19**, 132–146. 10.1038/s41571-021-00560-7 (2022). 34663898 10.1038/s41571-021-00560-7 PMC9034765

[11] Peeken, J. C., Nusslin, F. & Combs, S. E. Radio-oncomics: The potential of radiomics in radiation oncology. *Strahlenther. Onkol.* **193**, 767–779. 10.1007/s00066-017-1175-0 (2017). 28687979 10.1007/s00066-017-1175-0

[12] Fox, M. J., Gibbs, P. & Pickles, M. D. Minkowski functionals: An MRI texture analysis tool for determination of the aggressiveness of breast cancer. *J. Magn. Reson. Imaging* **43**, 903–910. 10.1002/jmri.25057 (2016). 26453892 10.1002/jmri.25057

[13] Feng, Q., Hu, Q., Liu, Y., Yang, T. & Yin, Z. Diagnosis of triple negative breast cancer based on radiomics signatures extracted from preoperative contrast-enhanced chest computed tomography. *BMC Cancer* **20**, 579. 10.1186/s12885-020-07053-3 (2020). 32571245 10.1186/s12885-020-07053-3 PMC7309976

[14] Aristei, C. *et al.* Personalization in modern radiation oncology: Methods, results and pitfalls. Personalized interventions and breast cancer. *Front. Oncol.* **11**, 616042 (2021). 33816246 10.3389/fonc.2021.616042 PMC8012886

[15] Hacking, S. M., Yakirevich, E. & Wang, Y. From immunohistochemistry to new digital ecosystems: A state-of-the-art biomarker review for precision breast cancer medicine. *Cancer* **14**, 3469. 10.3390/cancers14143469 (2022). 10.3390/cancers14143469 PMC9315712 35884530

[16] Yamamoto, S., Maki, D. D., Korn, R. L. & Kuo, M. D. Radiogenomic analysis of breast cancer using MRI: A preliminary study to define the landscape. *Am. J. Roentgenol.* **199**, 654–663. 10.2214/AJR.11.7824 (2012). 22915408 10.2214/AJR.11.7824

[17] Dijk, L. V. *et al.* Parotid gland fat related magnetic resonance image biomarkers improve prediction of late radiation-induced xerostomia. *Radiother. Oncol.* **128**, 459–466. 10.1016/j.radonc.2018.06.012 (2018). 29958772 10.1016/j.radonc.2018.06.012 PMC6625348

[18] Llorián-Salvador, Ó. *et al.* The importance of planning ct-based imaging features for machine learning-based prediction of pain response. *Sci. Rep.* **13**, 17427. 10.1038/s41598-023-43768-6 (2023). 37833283 10.1038/s41598-023-43768-6 PMC10576053

[19] Kraus, K. M., Oreshko, M., Bernhardt, D., Combs, S. E. & Peeken, J. C. Dosiomics and radiomics to predict pneumonitis after thoracic stereotactic body radiotherapy and immune checkpoint inhibition. *Front. Oncol.* **13**, 1124592 (2023). 37007119 10.3389/fonc.2023.1124592 PMC10050584

[20] Saadatmand, P. *et al.* A dosiomics model for prediction of radiation-induced acute skin toxicity in breast cancer patients: Machine learning-based study for a closed bore Linac. *Eur. J. Med. Res.* **29**, 282. 10.1186/s40001-024-01855-y (2024). 38735974 10.1186/s40001-024-01855-y PMC11089719

[21] Avanzo, M. *et al.* Electron density and biologically effective dose (BED) radiomics-based machine learning models to predict late radiation-induced subcutaneous fibrosis. *Front. Oncol.*10.3389/fonc.2020.00490 (2020). 32373520 10.3389/fonc.2020.00490 PMC7186445

[22] Isaksson, L. J. et al. Machine Learning-Based Models for Prediction of Toxicity Outcomes in Radiotherapy. *Frontiers in Oncology 10*, 790 (2020). 32582539 10.3389/fonc.2020.00790 PMC7289968

[23] Lilla, C. *et al.* Predictive factors for late normal tissue complications following radiotherapy for breast cancer. *Breast Cancer Res. Treat.* **106**, 143–150. 10.1007/s10549-006-9480-9 (2007). 17221151 10.1007/s10549-006-9480-9

[24] Kole, A. J., Kole, L. & Moran, M. S. Acute radiation dermatitis in breast cancer patients: Challenges and solutions. *Breast Cancer (Dove Med. Press)* **9**, 313–323. 10.2147/BCTT.S109763 (2017). 28503074 10.2147/BCTT.S109763 PMC5426474

[25] Huang, C. J. *et al.* RTOG, CTCAE and WHO criteria for acute radiation dermatitis correlate with cutaneous blood flow measurements. *Breast* **24**, 230–236. 10.1016/j.breast.2015.01.008 (2015). 25777626 10.1016/j.breast.2015.01.008

[26] Fedorov, A. *et al.* 3D slicer as an image computing platform for the quantitative imaging network. *Magn. Reson. Imaging* **30**, 1323–1341. 10.1016/j.mri.2012.05.001 (2012). 22770690 10.1016/j.mri.2012.05.001 PMC3466397

[27] van Griethuysen, J. J. M. *et al.* Computational radiomics system to decode the radiographic phenotype. *Cancer Res.* **77**, e104–e107. 10.1158/0008-5472.CAN-17-0339 (2017). 29092951 10.1158/0008-5472.CAN-17-0339 PMC5672828

[28] Peng, H., Long, F. & Ding, C. Feature selection based on mutual information criteria of max-dependency, max-relevance, and min-redundancy. *IEEE Trans. Pattern Anal. Mach. Intell.* **27**, 1226–1238. 10.1109/TPAMI.2005.159 (2005). 16119262 10.1109/TPAMI.2005.159

[29] Lemaître, G., Nogueira, F. & Aridas, C. K. Imbalanced-learn: A python toolbox to tackle the curse of imbalanced datasets in machine learning. *J. Mach. Learn. Res.* **18**, 1–5 (2017).

[30] Jr, D. W. H. & Lemeshow, S. Applied Logistic Regression. (Wiley, , UK, 2004).

[31] Brancato, V., Cerrone, M., Lavitrano, M., Salvatore, M. & Cavaliere, C. A systematic review of the current status and quality of radiomics for glioma differential diagnosis. *Cancers (Basel)* **14**, 2731. 10.3390/cancers14112731 (2022). 35681711 10.3390/cancers14112731 PMC9179305

[32] Tibshirani, R. Regression shrinkage and selection via the Lasso. *J. Royal Stat. Soc. Ser. B (Methodological)* **58**, 267–288 (1996). 10.1111/j.2517-6161.1996.tb02080.x

[33] Cortes, C. & Vapnik, V. Support-vector networks. *Mach. Learn.* **20**, 273–297. 10.1007/BF00994018 (1995). 10.1007/BF00994018

[34] Breiman, L. Random forests. *Mach. Learn.* **45**, 5–32. 10.1023/A:1010933404324 (2001). 10.1023/A:1010933404324

[35] Pedregosa, F. *et al.* Scikit-learn: Machine learning in Python. *J. Mach. Learn. Res.* **12**, 2825–2830 (2011).

[36] Ludwig, C. G., Lauric, A., Malek, J. A., Mulligan, R. & Malek, A. M. Performance of radiomics derived morphological features for prediction of aneurysm rupture status. *J. NeuroInterventional Surg.* **13**, 755–761. 10.1136/neurintsurg-2020-016808 (2021). 10.1136/neurintsurg-2020-016808 33158993

[37] Trinh, D. L., Kim, S. H., Yang, H. J. & Lee, G. S. The efficacy of shape radiomics and deep features for glioblastoma survival prediction by deep learning. *Electronics* **11**, 1038. 10.3390/electronics11071038 (2022). 10.3390/electronics11071038

[38] Yap, F. Y. *et al.* Shape and texture-based radiomics signature on CT effectively discriminates benign from malignant renal masses. *Eur. Radiol.* **31**, 1011–1021. 10.1007/s00330-020-07158-0 (2021). 32803417 10.1007/s00330-020-07158-0

[39] Hatt, M. *et al.* 18F-FDG PET uptake characterization through texture analysis: Investigating the complementary nature of heterogeneity and functional tumor volume in a multi–cancer site patient cohort. *J. Nucl. Med.* **56**, 38–44. 10.2967/jnumed.114.144055 (2015). 25500829 10.2967/jnumed.114.144055

[40] Welch, M. L. *et al.* Vulnerabilities of radiomic signature development: The need for safeguards. *Radiother. Oncol.* **130**, 2–9. 10.1016/j.radonc.2018.10.027 (2019). 30416044 10.1016/j.radonc.2018.10.027

[41] van Timmeren, J. E., Cester, D., Tanadini-Lang, S., Alkadhi, H. & Baessler, B. Radiomics in medical imaging—How-to guide and critical reflection. *Insights Imaging* **11**, 91. 10.1186/s13244-020-00887-2 (2020). 32785796 10.1186/s13244-020-00887-2 PMC7423816

[42] Zhang, W., Guo, Y. & Jin, Q. Radiomics and its feature selection: A review. *Symmetry* **15**, 1834. 10.3390/sym15101834 (2023). 10.3390/sym15101834

[43] Haviland, J. S. *et al.* The UK standardisation of breast radiotherapy (START) trials of radiotherapy hypofractionation for treatment of early breast cancer: 10-year follow-up results of two randomised controlled trials. *Lancet Oncol.* **14**, 1086–1094. 10.1016/S1470-2045(13)70386-3 (2013). 24055415 10.1016/S1470-2045(13)70386-3

