# Supplementary material for: CT-based radiomics for predicting breast cancer radiotherapy side effects
Source: Sci Rep. 2024 Aug 29;14:20051. doi: 10.1038/s41598-024-70723-w (PMC11362146; doi:10.1038/s41598-024-70723-w)
Supplement: Supplementary file 1 — Supplementary Information. [file 41598_2024_70723_MOESM1_ESM.docx]

**Supplemental Material**

[Supplemental Tables 2](#_Toc173133707)

[TABLE S1 2](#_Toc173133708)

[TABLE S2 2](#_Toc173133709)

[TABLE S3 2](#_Toc173133710)

[TABLE S4 3](#_Toc173133711)

[TABLE S5 3](#_Toc173133712)

[TABLE S6 4](#_Toc173133713)

[TABLE S7 4](#_Toc173133714)

[TABLE S8 4](#_Toc173133715)

[Supplemental Figures 6](#_Toc173133716)

[FIGURE S1 6](#_Toc173133717)

[FIGURE S2 6](#_Toc173133718)

[FIGURE S3 6](#_Toc173133719)

[FIGURE S4 7](#_Toc173133720)

[FIGURE S5 8](#_Toc173133721)

# Supplemental Tables

## TABLE S1

**Side effect prediction extended scores**

**Table S1.** Acquisition parameters extracted from the DICOM metadata files of the TUM center.

| **Acquisition parameters** | Matrix (pixel) | Pixel Spacing (mm) | Slice Thickness (mm) | Kernel | Tube Current (kV) | CT Scanner |
| --- | --- | --- | --- | --- | --- | --- |
| **TUM** | 512 x 512 | 0.97 x 0.97 | 3 | B31s | 130 | Siemens Somatom Emotion 16 |

## TABLE S2

**Side effect prediction extended scores**

**Table S2.** Test scores of the best performing models for each of the side effects and their configuration. For instance, the best model that predicted skin inflammation was a RF trained on the TBV cohort, which used Spearman’s correlation as the feature selection technique.

| **Metric** | **Moist Cells Epitheliolysis** | **Edema** | |  |
| --- | --- | --- | --- | --- |
|  | TBV, LASSO, MRMR | GT, LASSO, Spearman | |  |
| **AUROC*** | 0.74 ± 0.01 | | 0.55 ± 0.01 | |
| **Balanced Accuracy** | 0.65 | | 0.52 | |
| **F1** | 0.35 | | 0.15 | |
| **Sensitivity** | 0.56 | | 0.31 | |
| **Specificity** | 0.75 | | 0.73 | |
| **MCC** | 0.25 | | 0.03 | |

* Data is given as mean ± 1.96 standard errors for a 95% confidence interval

## TABLE S3

**Best radiomics cohort extended scores**

**Table S3.** Test scores of the best performing models depending on the training data. For instance, for the radiomics cohort TBV, a LASSO classifier performed best when using MRMR as the feature selection technique, and predicting moist cells epitheliolysis.

| **Metric** | **TBV** | **GT** | **Clinical Features** |
| --- | --- | --- | --- |
|  | Moist ep., LASSO, MRMR | Moist ep., RF, MRMR | Moist ep., LR, Spearman |
| **AUROC** | 0.74 ± 0.01 | 0.65 ± 0.01 | 0.70 ± 0.01 |
| **Balanced Accuracy** | 0.65 | 0.59 | 0.65 |
| **F1** | 0.35 | 0.27 | 0.34 |
| **Sensitivity** | 0.56 | 0.46 | 0.57 |
| **Specificity** | 0.75 | 0.71 | 0.71 |
| **MCC** | 0.25 | 0.14 | 0.23 |

* Data is given as mean ± 1.96 standard errors for a 95% confidence interval

## TABLE S4

**Best modelling strategy**

The four ML algorithms have been compared, and their best configurations of prediction target, radiomics feature set and feature selection technique are shown. The best performing ML algorithm was a LASSO classifier trained on TBV radiomics features to predict moist epitheliolysis (AUROC of 0.74), albeit not by a statistically significant margin: LR and RF are still within 1.96 standard errors at 0.73 and 0.72, respectively. Regardless of the algorithm selected, the configuration that has proven to perform best was using TBV as the training radiomics features, and MRMR as the feature selection technique. Moist epitheliolysis as the prediction target has, again, proven to yield the best results.

**Table S4.** Test scores of the best performing model configurations for each of the four ML algorithms. For instance, the best LR performance was achieved when trained on volume A radiomics features, using MRMR as the feature selection technique, and predicting moist cells epitheliolysis.

| **Metric** | **LR** | **LASSO** | **SVM** | **RF** |
| --- | --- | --- | --- | --- |
|  | TBV, Moist ep., MRMR | TBV, Moist ep., MRMR | TBV, Moist ep., MRMR | TBV, Moist ep., MRMR |
| **AUROC** | 0.73 ± 0.01 | 0.74 ± 0.01 | 0.69 ± 0.02 | 0.72 ± 0.01 |
| **Balanced Accuracy** | 0.65 | 0.65 | 0.63 | 0.64 |
| **F1** | 0.34 | 0.35 | 0.32 | 0.33 |
| **Sensitivity** | 0.56 | 0.56 | 0.47 | 0.5 |
| **Specificity** | 0.74 | 0.75 | 0.78 | 0.77 |
| **MCC** | 0.23 | 0.25 | 0.21 | 0.22 |

* Data is given as mean ± 1.96 standard errors for a 95% confidence interval

## TABLE S5

**Best feature selection approach**

The performance of the two different feature selection techniques is shown. Using either technique, LASSO performed best when training on the selected TBV features, and predicting moist cells epitheliolysis.

The impact of the feature selection technique is minimal, albeit noticeable, when compared to other modelling configurations, such as the prediction target or the ML algorithm. This can be seen not only for the best model overall (LASSO classifier, trained on the selected TBV radiomics features, and predicting moist cells epitheliolysis), but in many other instances. While MRMR has performed slightly better on the most optimal models, Spearman leads to marginally better results on just above random performing models.

**Table S5.** Test scores of the best performing models depending on the feature selection technique utilized. For instance, when using MRMR to select the best volume A radiomics features, LASSO performed best when predicting moist cells epitheliolysis.

| **Metric** | **MRMR** | **Spearman** |
| --- | --- | --- |
|  | TBV, Moist ep., LASSO | TBV, Moist ep., LASSO |
| **AUROC** | 0.74 ± 0.01 | 0.72 ± 0.01 |
| **Balanced Accuracy** | 0.65 | 0.64 |
| **F1** | 0.35 | 0.33 |
| **Sensitivity** | 0.56 | 0.5 |
| **Specificity** | 0.75 | 0.77 |
| **MCC** | 0.25 | 0.23 |

* Data is given as mean ± 1.96 standard errors for a 95% confidence interval

## TABLE S6

**Combined modelling extended results**

**Table S6.** Test scores of the best performing combined models for each of the radiomics cohorts. For instance, when using TBV radiomics features together with clinical features as training data, a RF classifier performed best when predicting skin inflammation.

| **Metric** | **TBV + Clinical Features** | | **GT + Clinical Features** | |
| --- | --- | --- | --- | --- |
|  | **Moist ep.** | **Edema** | **Moist ep.** | **Edema** |
|  | LASSO, MRMR | RF, Spearman | RF, MRMR | LASSO, Spearman |
| **AUROC** | 0.73 ± 0.01 | 0.53 ± 0.02 | 0.67 ± 0.01 | 0.55 ± 0.01 |
| **Balanced Accuracy** | 0.65 | 0.51 | 0.6 | 0.52 |
| **F1** | 0.34 | 0.12 | 0.29 | 0.15 |
| **Sensitivity** | 0.55 | 0.17 | 0.49 | 0.33 |
| **Specificity** | 0.74 | 0.86 | 0.71 | 0.71 |
| **MCC** | 0.23 | 0.03 | 0.16 | 0.02 |

* Data is given as mean ± 1.96 standard errors for a 95% confidence interval

## TABLE S7

**Best performing TBV model excluding volume-correlated features**

**Table S7.** Test scores of the best performing TBV model, excluding features with a Spearman correlation coefficient larger than 0.8 towards breast volume. The configuration was a LASSO classifier, using MRMR as the feature selection technique for further refinement, and predicting moist epitheliolysis.

| **Metric** | **TBV excluding volume features** |
| --- | --- |
|  | Moist ep., LASSO, MRMR |
| **AUROC** | 0.71 ± 0.01 |
| **Balanced Accuracy** | 0.63 |
| **F1** | 0.32 |
| **Sensitivity** | 0.51 |
| **Specificity** | 0.75 |
| **MCC** | 0.21 |

* Data is given as mean ± 1.96 standard errors for a 95% confidence interval

## TABLE S8

**Feature importance report of the best performing clinical model**

Table S8. Feature importance report of the best performing clinical model: a LR classifier trained on clinical features, selected with a double Spearman rank correlation test, and predicting moist cells epitheliolysis. Score is calculated by multiplying the average importance of a feature by their selection frequency. The best 15 features are shown.

| **Feature** | **% Selected** | **Average Importance** | **Score** |
| --- | --- | --- | --- |
| TBV Volume | 100 | 3.29 | 3.29 |
| RT Boost | 100 | 0.42 | 0.42 |
| EQD2 Max Radiation Dose | 100 | 0.31 | 0.31 |
| Smoker Status | 100 | 0.28 | 0.28 |
| Chemotherapy | 100 | 0.26 | 0.26 |

# Supplemental Figures

## FIGURE S1

**VOI references of CT scans for TBV (left) and GT (right)**


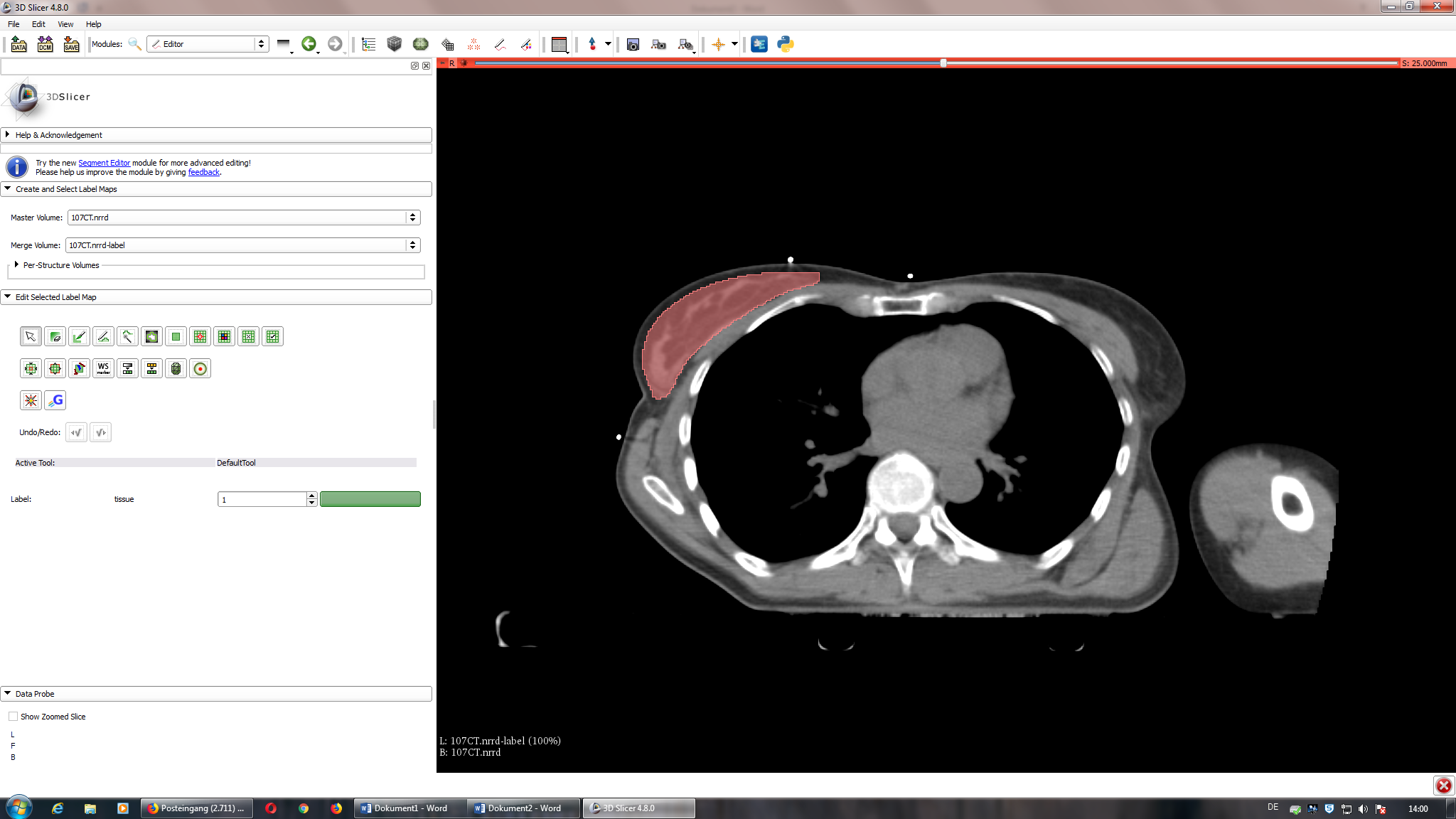

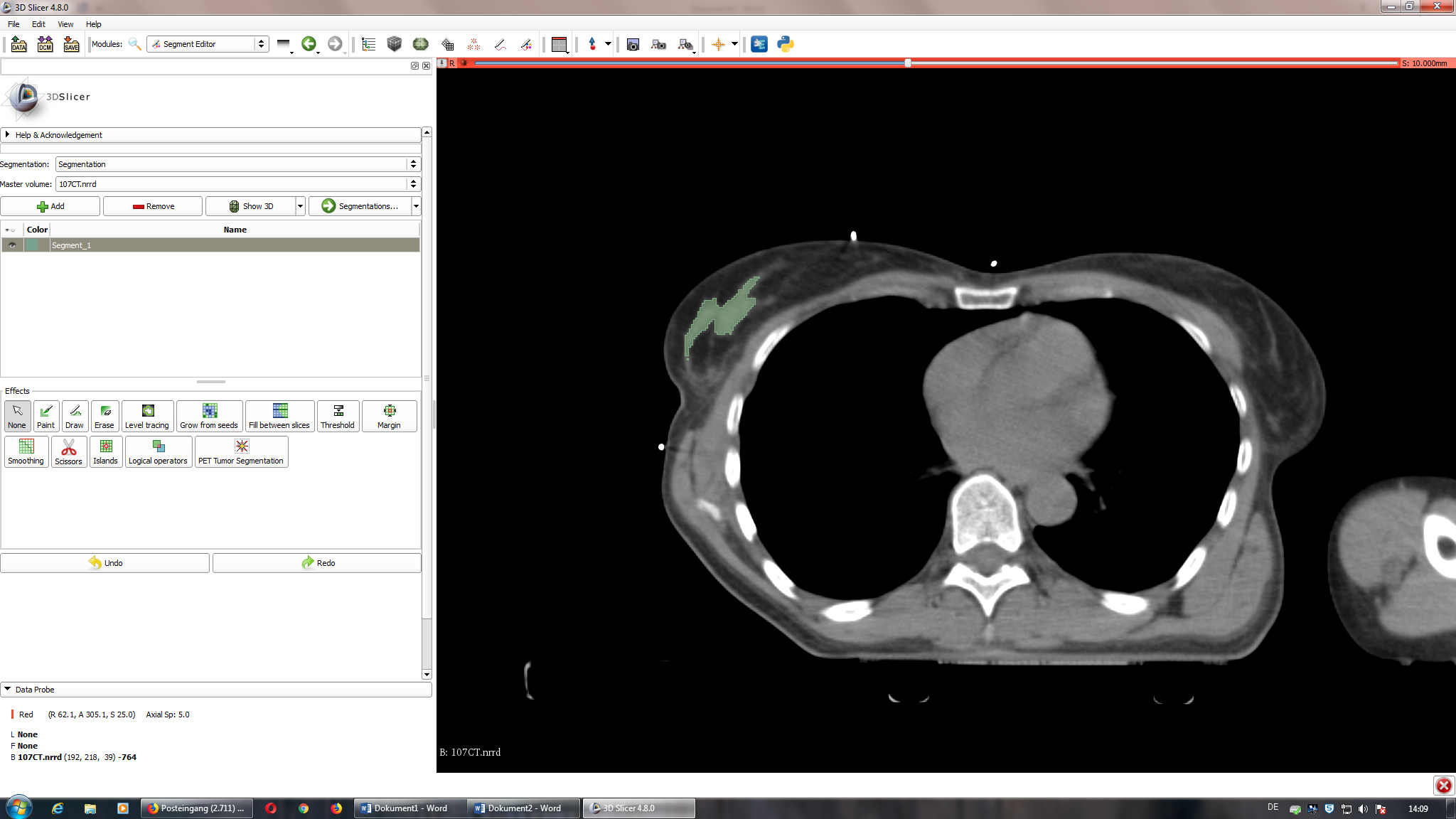


Figure S1. CT scans highlighting both VOIs studied in this research: TBV (left) and GT (right).

## FIGURE S2

**Patient workflow of clinical features and side effects**

**
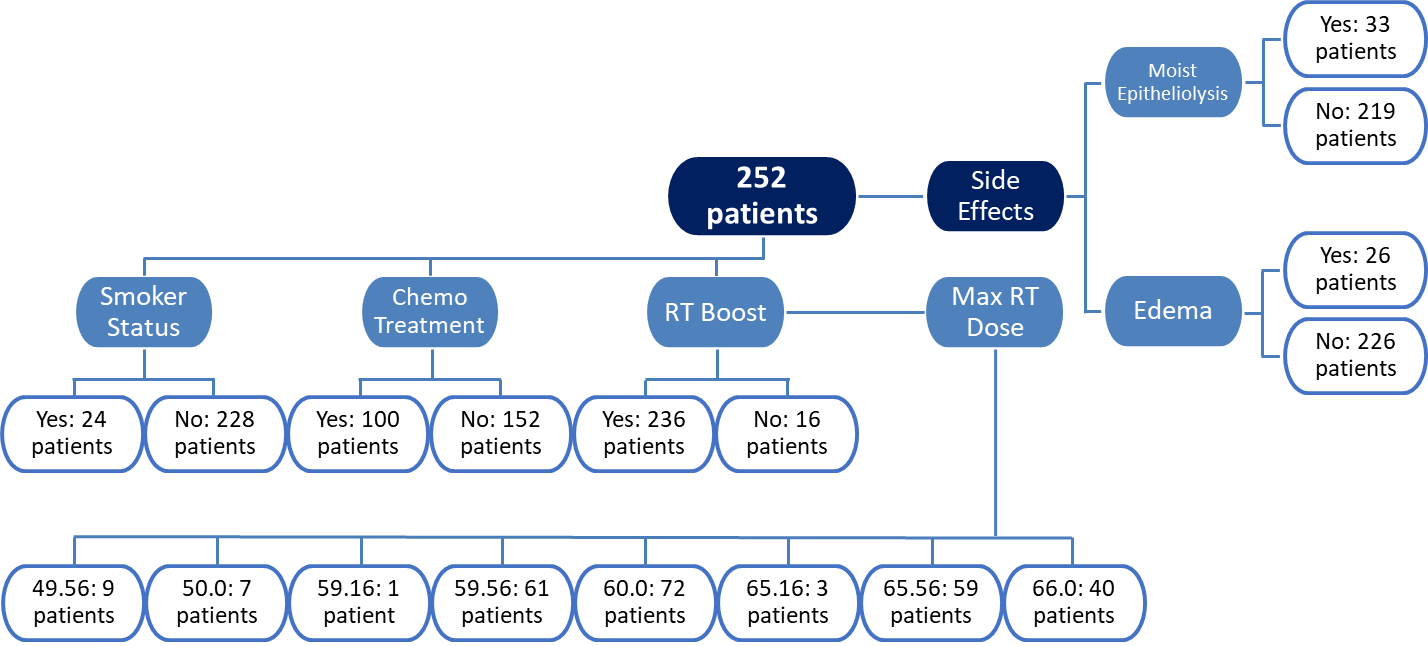
**

Figure S2. Patient workflow summarizing their clinical features and side effects distributions.

## FIGURE S3

**Extended predictive influence of the breast volume (correlation scores)**


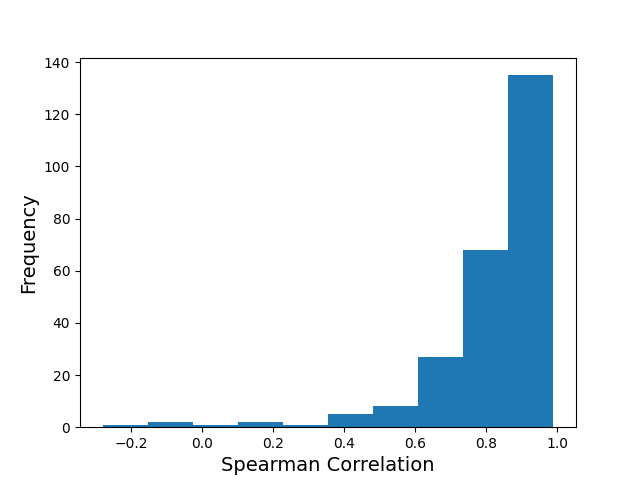


Figure S3. Spearman's correlation scores between the volume of the whole breast and the prediction probabilities of the best performing model, a LASSO classifier trained on TBV radiomics features, selected by MRMR, and used to predict moist cells epitheliolysis as a surrogate for skin inflammation side effect.

## FIGURE S4

**Extended predictive influence of the breast volume (correlation p-values)**


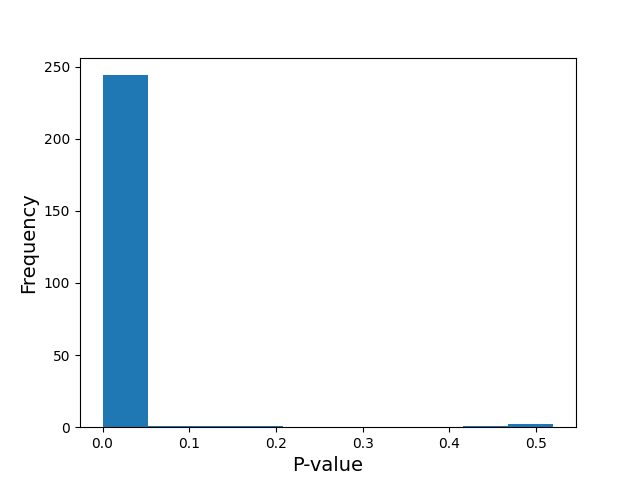


Figure S4. P-values of the Spearman's correlations between the volume of the whole breast and the prediction probabilities of the best performing model, a LASSO classifier trained on TBV radiomics features, selected by MRMR, and used to predict moist cells epitheliolysis as a surrogate for skin inflammation side effect.

## FIGURE S5

**Calibration curve of the best performing radiomics model**


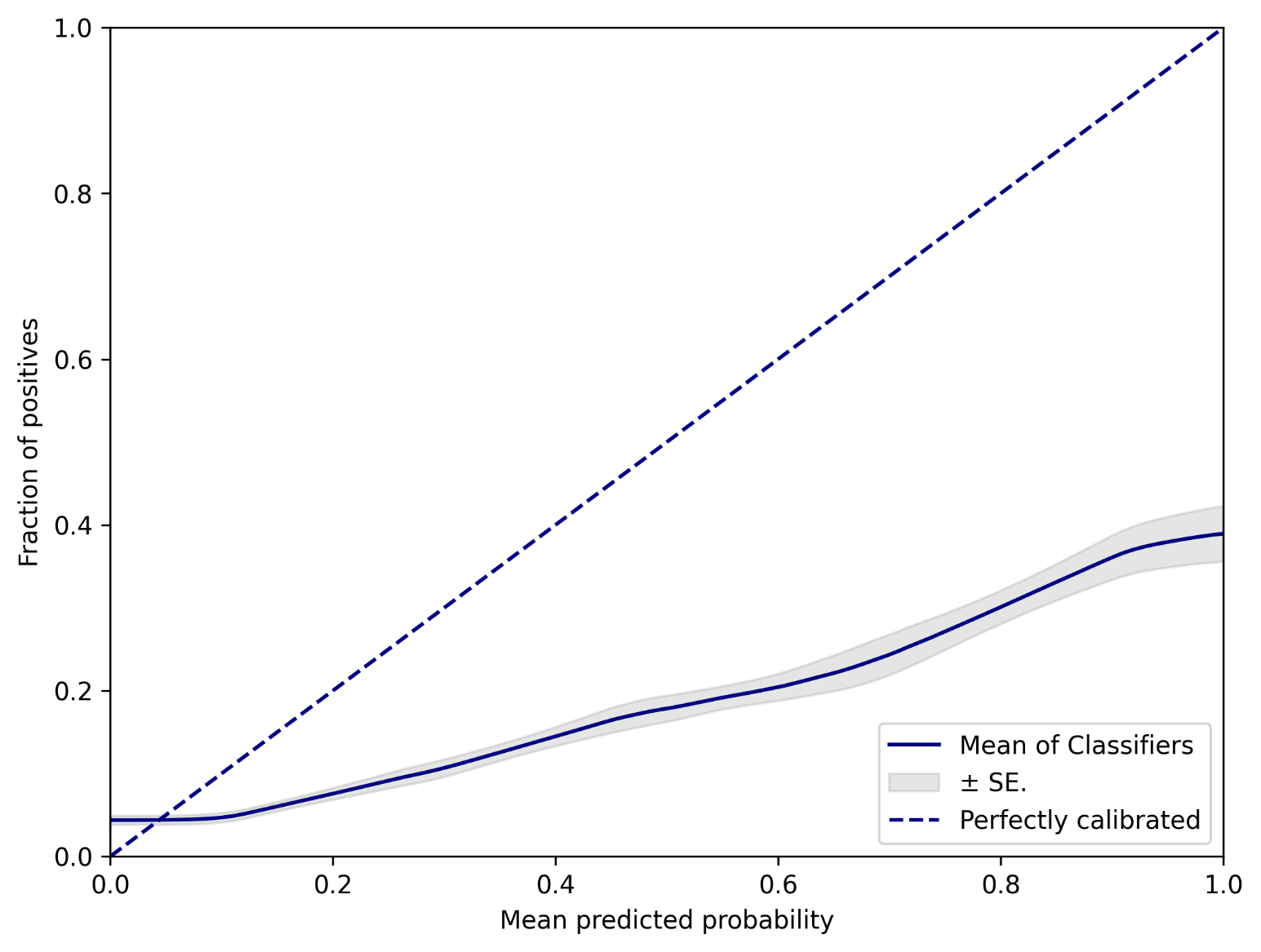


Figure S5. Calibration curve of the best performing radiomics model: a LASSO classifier trained on TBV radiomics features, selected with MRMR, and predicting moist cells epitheliolysis.
